# Genetically Encoded Biosensors for the Fluorescence Detection of O_2_ and Reactive O_2_ Species

**DOI:** 10.3390/s23208517

**Published:** 2023-10-17

**Authors:** Marialaura Marchetti, Luca Ronda, Monica Cozzi, Stefano Bettati, Stefano Bruno

**Affiliations:** 1 Department of Medicine and Surgery, University of Parma, 43125 Parma, Italy; marialaura.marchetti@unipr.it (M.M.); luca.ronda@unipr.it (L.R.); monica.cozzi@unipr.it (M.C.); 2 Institute of Biophysics, Italian National Research Council (CNR), 56124 Pisa, Italy; 3 Department of Food and Drug, University of Parma, 43124 Parma, Italy; stefano.bruno@unipr.it

**Keywords:** oxygen sensor, reactive oxygen species, fluorescent proteins, genetically encoded

## Abstract

The intracellular concentrations of oxygen and reactive oxygen species (ROS) in living cells represent critical information for investigating physiological and pathological conditions. Real-time measurement often relies on genetically encoded proteins that are responsive to fluctuations in either oxygen or ROS concentrations. The direct binding or chemical reactions that occur in their presence either directly alter the fluorescence properties of the binding protein or alter the fluorescence properties of fusion partners, mostly consisting of variants of the green fluorescent protein. Oxygen sensing takes advantage of several mechanisms, including (i) the oxygen-dependent hydroxylation of a domain of the hypoxia-inducible factor-1, which, in turn, promotes its cellular degradation along with fluorescent fusion partners; (ii) the naturally oxygen-dependent maturation of the fluorophore of green fluorescent protein variants; and (iii) direct oxygen binding by proteins, including heme proteins, expressed in fusion with fluorescent partners, resulting in changes in fluorescence due to conformational alterations or fluorescence resonance energy transfer. ROS encompass a group of highly reactive chemicals that can interconvert through various chemical reactions within biological systems, posing challenges for their selective detection through genetically encoded sensors. However, their general reactivity, and particularly that of the relatively stable oxygen peroxide, can be exploited for ROS sensing through different mechanisms, including (i) the ROS-induced formation of disulfide bonds in engineered fluorescent proteins or fusion partners of fluorescent proteins, ultimately leading to fluorescence changes; and (ii) conformational changes of naturally occurring ROS-sensing domains, affecting the fluorescence properties of fusion partners. In this review, we will offer an overview of these genetically encoded biosensors.

## 1. Relevance of O_2_ and ROS Sensing

The evolution of photosynthesis and the consequent oxygenation of the Earth’s atmosphere about 2.4 billion years ago is one of the most relevant transitions in the history of Life. A minority of organisms have since evolved in anoxic environments. Others have evolved in contact with O_2_, developing biochemical mechanisms and pathways to manage its toxicity and use it as the final acceptor of electrons in oxidative metabolism and as a substrate in several other reactions [1]. In these organisms, the availability of O_2_ is a key factor in their survival, and hypoxia, which hinders O_2_-dependent metabolic reactions, is involved in disease. In humans, hypoxia has been correlated with multiple sclerosis, cancer, heart disease, kidney disease, liver disease, lung disease, and inflammatory bowel disease [2]. To accurately measure O_2_ concentration inside cells and gain insights into these physiological and pathological conditions, O_2_ imaging is becoming increasingly important in cell biology. Among the available approaches, genetically encoded probes are the most relevant, since they allow the monitoring of O_2_ in real-time, in single cells, and without altering their physiology [3,4].

The great advantage that O_2_ has afforded to Life is counterbalanced by its toxicity. Indeed, O_2_ can undergo reactions that produce reactive O_2_ species (ROS), including hydrogen peroxide (H_2_O_2_), superoxide (O^2−^), singlet O_2_ (^1^O_2_), and hydroxyl radical (^●^OH). The generation of ROS is a double-edged sword. On the one hand, ROS mediate fundamental processes such as cellular signaling and immune response. On the other hand, their high reactivity can damage biological macromolecules. Therefore, monitoring ROS production in vivo allows the study of physiological reactions as well as cellular oxidative stress and the progression of diseases. As for O_2_, genetically encoded probes are the tool of choice. Due to their reactivity and the action of specialized enzymes such as superoxide dismutase, the half-life times of ROS are very short, making their direct detection challenging; therefore, the existing genetically encoded fluorescent ROS biosensors rely on the monitoring of H_2_O_2_ or the ROS effect on other redox-sensitive molecules, such as glutathione, thioredoxins (Trx), methionine, nicotinamide adenine dinucleotide, and its phosphate form. In particular, we will focus the discussion on those sensors able to directly measure the cellular H_2_O_2_ fluctuations based on the variation of the reduced/oxidized glutathione (GSH/GSSG) or Trx redox couples. Overall, the detection of ROS in vivo is notoriously biased and can lead to misleading claims. A consensus statement was recently published to clarify the limits of commonly used approaches and to propose guidelines for best practice [5].

Genetically encoded tools employed for the detection of O_2_ and ROS are typically based on fluorescence. Fluorescence has several advantages over other spectroscopic and microscopic techniques in biomolecular and cellular studies. This holds in terms of signal specificity, since fluorophores are excited and emit light at characteristic wavelengths, and background signal rejection, especially when two- or multi-photon excitation is exploited. Moreover, fluorescence is endowed with high sensitivity to physicochemical conditions of the microenvironment, since emission intensity and the Stokes shift markedly depend on solvent polarity, temperature and possibly pH, redox state and the presence of quenchers, and to structural dynamics. Indeed, since fluorescence is a relatively slow process, occurring on a time-scale of 10^−9^ s, it is more sensitive than other spectroscopic techniques, such as absorbance and circular dichroism, to local conformational changes of macromolecules, rotational and vibrational transitions, and protonation and deprotonation reactions.

Among fluorescent probes, fluorescent proteins (FPs) represent the most frequently used tool for several genetically encoded biosensors. They constitute a large family of homologous proteins from coral or other marine organisms. The most famous FP is the *Aequorea victoria* green fluorescent protein (GFP) [6]. In general, green fluorescence represents a limit in imaging techniques applied to deep tissues because of the scarce penetrating capacity of visible light. Wavelengths longer than 500 nm can reach deeper regions in in vivo experiments, also demonstrating lower phototoxicity (lower energy) and background absorption and autofluorescence (due to the presence of biomolecules such as melanin and hemoglobin). Therefore, natural or non-natural FP variants exhibiting different fluorescence properties have been produced. They all exhibit an intrinsic chromophore, which forms upon an internal, autocatalytic, and post-translational modification involving three consecutive amino acids (e.g., Ser65-Tyr66-Gly67 in the prototypical *A. victoria* wild-type GFP). Among the better-characterized variants relevant to this review are: (i) enhanced green fluorescent protein (EGFP) [7], created by introducing mutations to the original GFP gene to enhance its brightness; (ii) yellow fluorescent protein (YFP) [8], emitting yellow fluorescence, which was developed by introducing mutations into the GFP sequence, shifting its emission wavelength towards the yellow spectrum. YFP is often used in combination with other fluorescent proteins to perform fluorescence resonance energy transfer (FRET) experiments; (iii) cyan fluorescent protein (CFP) [7], a GFP variant that emits cyan fluorescence. Like YFP, CFP is used in FRET studies; (iv) red fluorescent protein (RFP) [9], a GFP variant that emits red fluorescence. It was engineered by introducing additional mutations to shift the emission wavelength towards the red spectrum. RFP is particularly useful for multicolor labeling and tracking of multiple proteins or cellular structures simultaneously.

In this review, we will focus on FPs designed for O_2_ and ROS detection.

## 2. Biosensors for O_2_

### 2.1. O_2_ Sensing Mediated by Prolyl Hydroxylases

**Figure 1:**
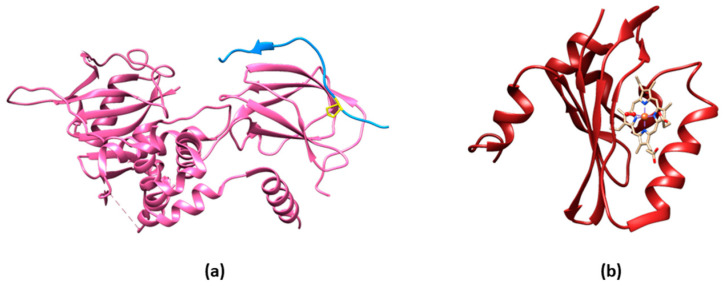
Structures of O_2_ sensing proteins. (**a**) Crystal structure of a hydroxylated HIF-1 α peptide (in blue)—with the hydroxylated proline represented in yellow sticks—bound to the human pVHL/elongin-C/elongin-B complex (in pink) (PDB ID 1lqb) [17]. (**b**) Crystal structure of the *Escherichia coli* sensor heme domain (Ec DosH). The oxy heme is represented in sticks (PDB ID 1s66) [18].

The transcription factor hypoxia-inducible factor-1 (HIF-1) is the main actor in modulating O_2_-mediated gene expression levels in all Parahoxozoa. HIF-1 is a *α*β heterodimer with the levels of the β subunit being O_2_-independent, whereas those of the α subunit are O_2_-dependent [10] through its O_2_-dependent proteolysis [11]. In fact, the α subunit is constitutively synthesized but rapidly degraded under normoxic conditions [12] as a consequence of enzymatic hydroxylation of conserved proline residues (Pro402 and Pro564) located in its oxygen-dependent degradation domain (ODD) [13,14]. The enzymes responsible for proteolysis are 2-oxoglutarate-dependent dioxygenases containing a prolyl hydroxylase domain (PHD) [15]. Hydroxylated HIF-1α binds to the von Hippel–Lindau tumor suppressor protein (VHL) and is then ubiquitylated by the VHL-E3 ligase complex (Figure 1a). Ubiquitination directs HIF-1α to degradation by the 26S proteasome [16]. The 2-oxoglutarate-dependent dioxygenases use dioxygen to hydroxylate proline residues, thus constituting the link between O_2_ concentration, HIF-1α degradation, and HIF-1α-mediated gene transcription.

**Figure 2:**
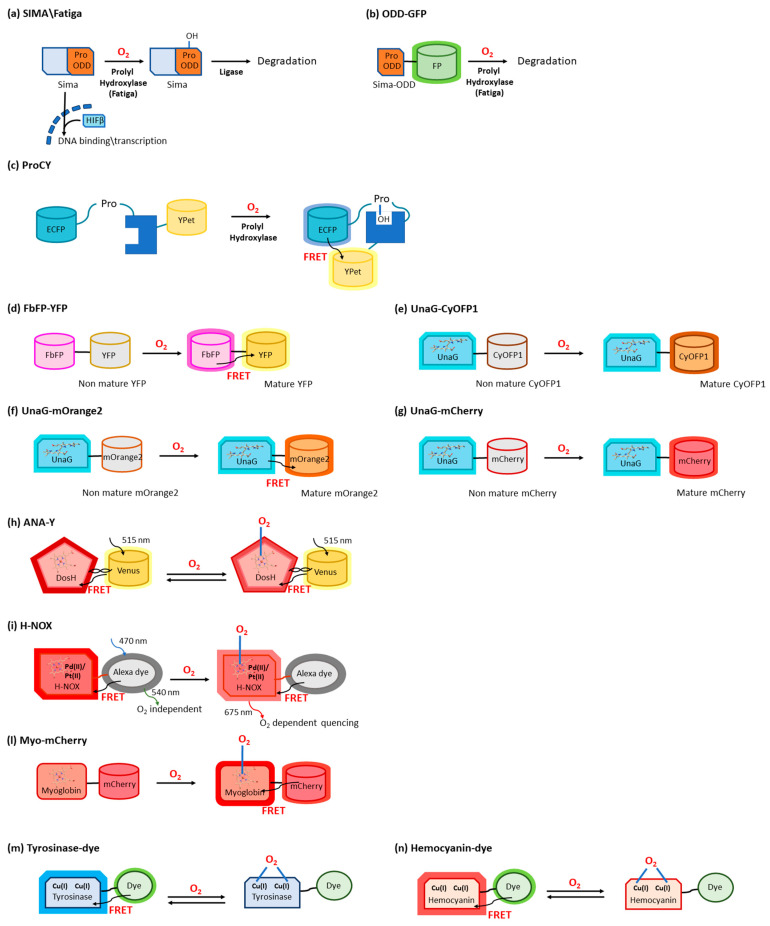
Schematic representation of selected O_2_ sensors. (**a**) Mechanism of O_2_ sensing by Sima. (**b**) Fusion protein between Sima ODD domain and FPs. (**c**) Mechanism of O_2_ sensing by the ProCY fusion protein. (**d**) Mechanism of O_2_ sensing by maturation-dependent FbFP. The hypoxia-tolerant fluorescent protein UnaG fused with: (**e**) CyOFP1 FP for the development of ratiometric reporter; (**f**) mOrange2 FP for the development of FRET-FLIM reporter; and (**g**) mCherry FP for the development of ratiometric reporter. The UnaG bilirubin cofactor is represented in sticks. (**h**) O_2_ sensing by ANA-Y biosensor; DosH heme cofactor is represented in sticks. (**i**) O_2_ sensor based on H-NOX; the porphyrin cofactor is represented in sticks. (**l**) mCherry fused with myoglobin as an O_2_ sensor; myoglobin heme cofactor is represented in sticks. (**m**) Mechanism of tyrosinase copper protein conjugated with a fluorescent dye to sense O_2_. (**n**) Mechanism of hemocyanin copper protein conjugated with a fluorescent dye to sense O_2_.

A protein homologous to HIF-1α from *Drosophila melanogaster*, called Sima (bHLH/PAS domain transcription factor Similar), follows a fate similar to HIF-1α: Fatiga, a prolyl hydrolase, hydroxylates Pro-850 [19] on the Sima O_2_-dependent domain. Under hypoxic conditions, lower O_2_ availability limits proline hydroxylation. Non-hydroxylated Sima accumulates, translocates into the nucleus, and heterodimerizes with HIF β (Tango) for binding to a hypoxic response element (HRE) (Figure 2a) [20].

The pathway of HIF-1α and Sima has been considered as a suitable sensor for O_2_. For example, a biosensor based on Sima is composed of a fusion protein including its ODD (692–863 residues) and the green fluorescent protein (GFP) (Figure 2b) [21]. The fusion construct (GFP-ODD) is constitutively expressed under the control of the ubiquitin-69E (ubi) promoter. Thanks to the GFP fluorescence emission, local differences among different tissues and cells within the same tissue in hypoxic or normoxic conditions can be followed in vivo. Moreover, to follow even small changes in O_2_ concentration, GFP-ODD has been co-expressed with monomeric red FP with a nuclear localization signal (mRFP-nls). This latter protein expression is not affected by changes in O_2_ level and is used to normalize the GFP-ODD signal with respect to other factors affecting its level in an O_2_-independent manner. A ratiometric measurement between GFP-ODD and mRFP-nls only depends on a differential degradation of GFP-ODD mediated by Fatiga [22].

Another genetically encoded biosensor sensitive towards proline hydroxylation by PHD, called ProCY, has been developed for measuring O_2_ in vitro and in live mammalian cells. The protein is formed of a 22-amino acid peptide from HIF-1α (residues 556–577, containing Pro564) and a small 10-kDa protein domain derived from VHL (residues 60–154) sandwiched between two FPs—the enhanced cyan FP (ECFP) and a yellow FP YPet—that are a highly optimized FRET couple. Pro564 hydroxylation promotes the interaction between the peptide and the VHL domain, thus causing a conformational change that alters the distance between ECPFP and YPet, giving a measurable change in the FRET signal (Figure 2c) [23].

### 2.2. O_2_ Sensors Based on O_2_-Dependent Maturation of GFP Variants

The plethora of FP variants that have been devised over the years to tailor spectroscopic, photochemical, and photophysical properties achieve fluorescence through an O_2_-dependent “maturation process” (Figure 2d–g) [24]. The O_2_-dependence of the chromophore maturation and fluorescence is sometimes considered a limit to the applicability of FPs as reporter molecules, therefore new classes of O_2_-independent fluorescent reporters have been developed, based, e.g., on photosensory flavoproteins [25,26,27]. On the other hand, the fact that fluorescence is acquired only after spontaneous auto-oxidation and cyclization of the chromogenic tripeptide highlights the possibility of using it for intracellular O_2_ sensing, e.g., by exploiting GFP differential expression under the control of O_2_-responsive *E. coli* promoters [28,29], or by devising fusion proteins containing two fluorescent domains able to provide ratiometric O_2_ sensing upon FRET. For instance, a FRET-based O_2_ sensor was developed by Potzkei et al. [30] via a fusion protein between a GFP variant, the yellow FP (YFP), which requires O_2_ for maturation of the chromophore, and the hypoxia-tolerant flavin-binding FP (FbFP) (Figure 2d). Efficient energy transfer from the FbFP donor to the YFP acceptor only occurs in the presence of O_2_, so changes in the FbFP/YFP fluorescence ratio allows real-time monitoring of O_2_ concentration in the cytoplasm of *E. coli* living cells.

Lidsky et al. [31] reported that O_2_ availability during maturation of DsRed FP determines the ratio between a red and a green fluorescent isoform (under normoxic and hypoxic conditions, respectively). The red-to-green fluorescence ratio of a DsRed FT-based probe (nlsTimer) could be used for recording the history of oxygenation in model animals (*Drosophila* larvae), with single-cell resolution. The nlsTimer is sensitive to O_2_ concentrations within the biologically relevant range close to normoxia, and its use might in principle be extended to investigate hypoxia in other small laboratory animals; one of the main limits, according to the authors, resides in the slow irreversible maturation of DsRed preventing the detection of rapid changes in O_2_ availability.

Very recently, O_2_-dependent maturation of FPs (the orange large Stokes shift protein CyOFP1 and mOrange2) fused with the hypoxia-tolerant fluorescent protein UnaG was investigated for the development of ratiometric and FRET-fluorescent lifetime imaging microscopy (FLIM) reporters, respectively (Figure 2e,f) [32,33]. Different from most GFPs and red fluorescent proteins (RFPs), the GFP UnaG from Japanese eel [34] does not acquire fluorescence upon a relatively slow (lifetime from minutes to hours) O_2_-dependent maturation of a chromogenic triad [35] but upon O_2_-independent binding of a fluorogenic cofactor, the porphyrin metabolite bilirubin. In vitro studies exploiting the fusion proteins dUnORS (destabilized UnaG-CyOFP1 ratiometric sensor) and dUnOFLS (destabilized UnaG-mOrange2 fluorescence lifetime sensor) on spheroids and tumor transplantation models allowed to report O_2_ availability at cellular resolution [32]. Previously, a similar approach was applied to detect hypo-oxygenation and anoxia in plant tissues, through a fusion protein between UnaG and an O_2_-dependent RFP derived from DsRed, mCherry, under the control of an HRPE promoter (Figure 2g) [33]. The performance of the biosensor was optimized by testing the effect of different untranslated regions at the 5′ end of the reporter coding sequence. The sensor with the optimized promoter allowed direct imaging of different O_2_ concentrations, though in a reportedly narrow range, through a varying mCherry/UnaG ratiometric output. An ingenious method for in vivo O_2_ imaging using GFP is that proposed by Takahashi, Sato, and coworkers [36], exploiting the red shift of the fluorescence emitted by an enhanced GFP, occurring only under hypoxic conditions, to image hypoxia in COS-7 cultured cells or cardiomyocytes isolated from a GFP knock-in mouse heart.

### 2.3. O_2_ Sensors Based on O_2_-Binding Heme Proteins

Oxygen sensing has been also approached by exploiting heme-binding proteins, which use the iron-containing heme group as the O_2_ sensing element to be transduced in other cellular signals. These constructs are commonly composed of an N-terminal heme-containing globin domain, whose conformational variation upon O_2_ binding activates a functional domain that triggers a catalytic domain. This latter can have a diguanylate cyclase (DGC) [37] or phosphodiesterase (PDE) [38] activity toward cyclic diGMP (c-diGMP) and histidine kinase (HK) activity [39].

An example is a genetically encoded O_2_ biosensor based on the direct O_2_ sensor DosP from *E. coli*. This protein contains a heme-binding globin domain called DosH (Figure 1b) and a PDE catalytic domain, which converts cyclic-di-GMP to linear-di-GMP [40]. However, based on the crystallographic structures [18,41], the conformational variation of DosP upon O_2_ binding or dissociation is not sufficient to develop a suitable FRET signal; to overcome this limitation, DosH has been associated with a fluorescent protein to exploit the heme spectroscopic changes in Soret and Q peaks upon O_2_ binding shifting from 425 and 560 nm to 414 and 580 nm, respectively [42]. The yellow FP Venus—a GFP variant with a suitable spectral overlap with DosH—was conjugated with DosH using an optimized antiparallel coiled-coil linker. When Venus was excited at 500 nm, its fluorescence emission at 527 nm was absorbed by DosH in the O_2_-free form, resulting in low Venus quantum yield; when DosH was O_2_-bound, Venus’s fluorescence intensity increased. The change in heme absorption was therefore amplified by a change in fluorescence intensity. This sensor, called ANA-Y (anaerobic/aerobic sensor yellow), was used to sense O_2_ in the micromolar range (Figure 2h) [43].

The heme nitric oxide/oxygen-binding protein (H-NOX) of the thermophilic bacterium *Caldanaerobacter subterraneus* has been exploited to develop an O_2_ sensor as a robust protein scaffold also able to bind unnatural heme cofactors. The natural heme was replaced with a Pd(II) or Pt(II) porphyrin, a phosphorescent cofactor (650–800 nm range) that, in the triplet excited state, can interact with molecular oxygen; in particular, Pd(II) porphyrins are more sensitive to low O_2_ levels and Pd(II) has a larger range of O_2_ sensitivity. A ratiometric sensor based on H-NOX incorporating Pd(II) or Pt(II) porphyrins was developed by conjugating to the protein an Alexa fluorescent dye that is the O_2_-independent FRET donor to the porphyrin. The selective excitation of Alexa dye guarantees that the porphyrin emission will only be from FRET, thus minimizing the background signal (Figure 2i) [44].

As a different approach, the spectroscopic changes of heme upon O_2_ binding have been exploited and amplified in a fusion protein (Myo-mCherry), where myoglobin has been fused with mCherry fluorescent protein; spectral changes resulted in a change of FRET, captured as a change in fluorescence lifetime within cells by FLIM (Figure 2l). Different Myo-mCherry constructs have been prepared and tested, with two [45], one, four, and six [46]-residue linkers between the two proteins and with an H64Q mutation on myoglobin to reduce its oxygen affinity [46]. These variants allow to tune and adapt the dynamic range of this sensor in vitro to different cell lines and the construct can be targeted to subcellular compartments.

### 2.4. O_2_ Sensors Based on O_2_-Binding Copper Proteins

The use of type 3 copper proteins to sense O_2_ has been also approached; these proteins have a binding site with two Cu(I) ions that specifically bind to O_2_, and the complex, once formed, gives absorption peaks at 340 and 570 nm [47]. O_2_ binding typically also causes Trp fluorescence changes, but the use of fluorescence coming from an exogenous fluorophore with a more efficient and higher-wavelength emission is made necessary to increase sensitivity (Trp has a moderate quantum yield, around 0.14) and to allow the detection in tissues showing significant autofluorescence.

An example is given by the tyrosinase from the bacterium *Streptomyces antibioticus* and hemocyanin from the arthropod *Carcinus aestuarii*, which have been conjugated at the N-terminus with different fluorescent dyes (Alexa 350, Atto390, Cy3, Cy5, Atto655), showing an absorption overlapping Trp emission, thus allowing the detection as a variation in FRET upon O_2_ binding (Figure 2m) [48].

The detection of a single O_2_ molecule has been obtained by exploiting the O_2_ carrier hemocyanin from the tarantula *Eurypelma californicum*. This protein shows spectral differences when it binds oxygen, and these absorption changes can be used to sense O_2_ presence. Moreover, to amplify the signal, TAMRA fluorescent dye was conjugated with hemocyanin, and its fluorescence quantum yield was shown to decrease by a factor of two when hemocyanin was oxygenated [49]. This was caused by a FRET where TAMRA was the donor and the O_2_ binding site was the acceptor (Figure 2n) [50].

## 3. Biosensors for ROS

### 3.1. ROS Sensors Based on roFPs

**Figure 3:**
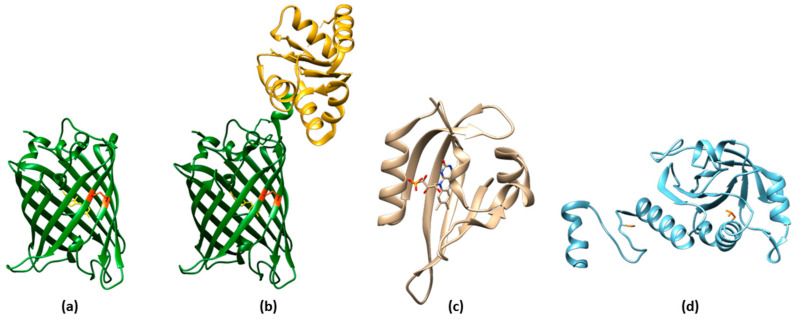
Structure of ROS sensing proteins. (**a**) Crystal structure of rxYFP in the oxidized form, with the reactive cysteine residues 149 and 202 in orange (PDB 1h6r) [51]. The fluorophore is represented in yellow. (**b**) YFP–glutaredoxin fusion protein (PDB ID 2jad) [52], with the YFP domain in green and the glutaredoxin domain in gold. The reactive cysteine residues 149 and 202 are shown in orange. The fluorophore is represented in yellow. (**c**) Crystal structure of the flavoprotein improved LOV (iLOV) domain (PDB ID 4eet) [53]. The FAD moiety is represented in sticks. (**d**) Crystal structure of human peroxiredoxin in the monomeric form (PDB ID 1qmv) [54]. The reactive cysteine residues (51 and 172) are represented in sticks.

**Figure 4:**
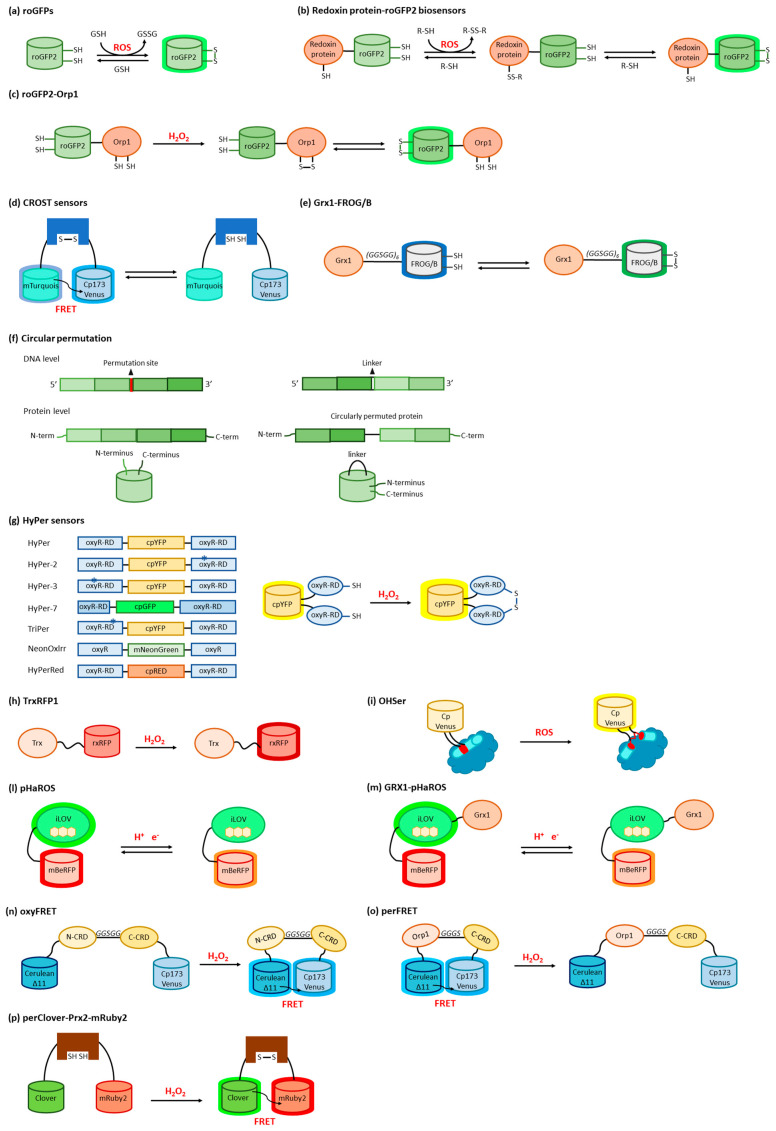
Schematic representation of selected ROS sensors. (**a**) General mechanism of roGFP-based biosensors. (**b**) Mechanism of biosensors based on a fusion protein between a roGFP2 and a redoxin protein. (**c**) Mechanism of roGFP-Orp1 biosensor. (**d**) Mechanism of CROST sensor. (**e**) Mechanism of Grx1-FROG/B sensor. (**f**) Representation of the circular permutation mechanism in proteins. (**g**) Schematic representation of HyPer sensor constructs and their general mechanism as ROS sensors; asterisks represent mutations inserted in the original HyPer construct. (**h**) Mechanism of TrxRFP1 sensor. (**i**) Mechanism of OHSer sensor. Mechanism of pHaROS (**l**) and of its variant GRX1-pHaROS (**m**). (**n**) Genetically encoded sensor for H_2_O_2_ based on the FRET couple Cerulean Δ11 and Cp173 Venus separated by the two CRDs of Yap1 (oxyFRET). (**o**) Genetically encoded sensor for H_2_O_2_ based on the FRET couple Cerulean Δ11 and Cp173 Venus separated by Orp1 linked with the C-terminal CRD of Yap1 (perFRET). (**p**) Mechanism of perClover-Prx2-mRuby2 biosensor.

Reduction-oxidation sensitive GFPs (roGFPs) are non-natural variants of *A. victoria* GFP obtained by substituting surface-exposed residues with cysteine residues appropriately distanced to form disulfide bonds at suitable redox potentials (Figure 3a,b and Figure 4a). The first examples of roFPs consisted of the introduction of an artificial pair of cysteine residues on YFP, giving rise to one of the first classes of redox-sensitive protein-based fluorescent sensors. The first rxYFP was conceived in 2001 by Winther’s research group, when the YFP sequence was modified to mutate Asn149 and Ser202 to two cysteine residues able to form a disulfide bridge under oxidizing conditions (Figure 3a) [51].

Cys149 and Cys202 are on β-strands 7 and 10, which are involved in shielding the chromophore from the surrounding environment; their position was chosen based on (i) the structural tolerance of modifications in this protein region and (ii) the ability of the disulfide bridge to reversibly perturb the protein structure and cause a modification in its fluorescence properties. One of the two wild-type YFP (wtYFP) cysteine residues, Cys48, was accordingly mutated to valine to avoid its potential reaction with the redox-sensing residues. The mutant YFP structure [51] superimposed very well that of wtYFP. The engineered protein maintained the spectral features of wtYFP, with two interconverting absorption peaks at 488 nm and 512 nm and one emission peak at 523 nm. Small rearrangements in the disulfide bond region and in the proximity of the chromophore, however, seemed to influence the chromophore position, leading to the reduction of its molar extinction coefficient, and its protonation state was likely affected, resulting in fluorescence decrease. The chromophore pK_a_ was also altered by the structural rearrangements evoked by the reduced-oxidized transition, making fluorescence strongly dependent on pH, especially at neutral values; therefore, the fluorescence dynamic range was found to be pH-sensitive. The biosensor functionality was also demonstrated in vivo in *E. coli* and *Saccharomyces cerevisiae* strains, where it equilibrated with the intracellular glutathione pool as the main reacting species [51,55]. An improved version of rxYFP was successively set up by the same researchers, with the introduction of three additional mutations in positions 200, 204, and 227, where the tyrosine, glutamine, and alanine were replaced with three arginine residues [56]. The presence of positive charges in these positions increased by thirteen-fold the reactivity of Cys149 towards GSSG by reducing its pK_a_, stabilizing the thiolate as a leaving group, and creating a favorable electrostatic environment for negatively charged reagents.

A further group of roGFP derivatives was based on the S147C, Q204C GFP variant on the C48S background (roGFP1), which involved residues adjacent to His148, which interacted with the chromophore phenolate oxygen [57]. As the resulting Cys147, Cys204 disulfide bond was relatively strained, the spectroscopic properties of the chromophore were altered in the oxidized form [58]. Particularly, the disulfide bond favored the protonation of the chromophore, thus increasing the excitation spectrum peak at 400 nm, while concomitantly decreasing that at 490 nm. Therefore, the ratiometric response to different redox environments—with a ratio increase up to six-fold in vitro [59]—correlated with the fraction of disulfide bond formation and, indirectly, with the surrounding redox potential. Ratiometric detection had the advantage of making the measurement insensitive to the expression levels [60]. Further mutations in the roGFP1 background (roGFP 2 to 6) produced a tunable response to the redox potential to suit the investigation of physiologically or pathologically relevant ranges [58]. Other than roGFP1, the most widely investigated roGFP is roGFP2, carrying the additional S65T mutation [58], known to increase the excitation efficiency at 480 nm and decrease that at 400 nm compared with wtGFP [8]. roGFP1 is more sensitive in the reduced range and is, therefore, more suitable for reducing cell compartments, such as mitochondria or the cytosol, whereas roGFP2 exhibits a larger dynamic range than roGFP1. The spectroscopic properties of roGFPs are altered by pH, but the intensity ratio at the excitation maxima is unaffected within physiological ranges, thus making roGFP-based measurements effectively pH-independent. 

When genetically encoded and suitably targeted to the desired cell compartment, the spectroscopic properties of roGFPs yield real-time information on the intracellular redox state of living cells. roGFP1 and roGFP2 were first used as probes of the redox environment of the matrix space and the cytosol of HeLa cells [58,59]. Several cell types and organisms have followed, including *Arabidopsis thaliana* [61,62,63], *Trypanosoma brucei* [64], coronary artery endothelial cells [65], human retinal endothelial cells [66], pulmonary arterial smooth muscle [67], cardiomyocytes [68], pancreatic islets and β-cells [69], and endothelial cells [70]. The well-known strategy to target GFP to cell compartments through its genetic fusion to signal peptides or proteins [71] was used to target roGFPs—among other compartments—to plant peroxisomes [62], plant mitochondria [72], *S. cerevisiae* mitochondria [73], the mammalian mitochondrial matrix (mito-roGFP, [74,75,76,77]), the mitochondrial intermembrane space (IMS-RoGFP, [74]), the mammalian ER [78], diatom chloroplasts (chl-roGFP, [79]), diatom mitochondria (GSIII) (mit-roGFP, [79]), and diatom nuclei (nuc-roGFP, [79]).

One limit of the original roGFP series lies in the slow response to changes in redox potential, thus making them unsuitable to follow rapid oxidative events. Moreover, the disulfide bond in the oxidized state is thermodynamically stable, limiting the applicability in the more oxidizing cell compartments as the endoplasmic reticulum. Variants of roGFPs have therefore been developed to address these issues. The substitution of positively charged amino acids in roGFP1 (roGFP1 R1-R14) substantially improved the response rate to changes in redox potential [80]. The roGFP1-iX family was produced from roGFP1 by introducing mutations 147CX and H148S in proximity to the disulfide bond, with the effect of reducing its stability and leading to midpoint potentials of −229 to −246 mV, better suited for the investigation of oxidizing subcellular compartments [78,81].

Another example of roGFPs was represented by rxmRuby2, a modified version of the mRuby2 probe, where Thr148 and Asp200 were mutated to cysteine to form a disulfide bridge when oxidized [82]. Under physiological conditions, rxmRuby2 is characterized by an excitation peak at 560 nm with a minor shoulder at 520 nm, resulting in an emission peak centered at 600 nm; the fluorescence intensity decreases upon redox cysteines oxidation, without ratiometric behavior.

### 3.2. ROS Sensors Based on roGFP Fusion Proteins

Studies on rxYFP expressed in yeast strains demonstrated that its oxidation is mediated by the sulfhydryl disulfide oxidoreductase glutaredoxins (Grxs) [55]. Based on this observation, Winther and colleagues exploited the catalytic activity of the yeast glutaredoxin 1 Grxp1 on rxYFP to design a fusion construct combining the two proteins (Figure 3b) [52,83]. rxYFP-Grx1p is glutathione-specific and does not rely on host Grxs when expressed in vivo, improving the sensor dynamic features and reaction rates and broadening its possible applications. Due to its redox potential of around −260 mV, rxYFP can find application for redox measurements in reducing cellular compartments but is not suited for reliable tests in secretory compartments [55].

Therefore, other fusion variants of roFPs have been developed. Particularly, Grx1-roGFP2 is a fusion protein of roGFP2 with Grx1 (Figure 4b). In comparison with roGFP, the equilibration rate with glutathione was higher by three orders of magnitude [84,85]. Grx1-roGFP2 allowed live imaging of the glutathione redox potential (EGSH) in several cell subcompartments, allowing for the detection of nanomolar changes in oxidized glutathione within seconds to minutes [85,86]. Grx1-roGFP2 was also used to probe thiol and redox metabolism in *Plasmodium falciparum* [87]. Several organelle- and cytoskeleton-targeted Grx1-roGFPs have been validated [88]. Fusion of roGFP with the NADPH oxidases (Nox) organizer protein p47 phox, a redox-sensitive protein that specifically reacts with Nox, was developed [89,90]. roGFP2-Orp1 is a fusion protein of roGFP2 with the *S. cerevisiae* protein oxidant receptor 1 (Orp1), a thiol-peroxidase that controls cellular H_2_O_2_ homeostasis by activating the transcription factor Yap1 by oxidation. The fusion protein with roGFP2 was specifically developed for the detection of H_2_O_2_ (Figure 4c) [84]. Indeed, it was shown that Orp1 is not restricted to the natural substrate Yap1 but can also oxidize its fusion partner roGFP in the fusion protein roGFP2-Orp1, thus constituting a probe for H_2_O_2_ [91]. roGFP2-Tpx1.C169S is a fusion protein with the C169S variant of *Schizosaccharomyces pombe* Tpx1. Tpx1 belongs to the group of peroxiredoxins (Prx), peroxidases that catalyze the reduction of H_2_O_2_. H_2_O_2_ first reacts with the so-called “peroxidatic” cysteine residue of Prxs to produce cysteine sulfenic acid, which then reacts with the “resolving” Cys residue to form a disulfide bond. Prxs can then be reduced by Trx by disulfide exchange. The Cys169 variant of Tpx1 interacts with H_2_O_2_, but the peroxidatic Cys48-SOH cannot react further with the resolving Cys169 [92]. The fusion proteins roGFP2-Tsa2ΔC_R_ and roGFP2-Tsa2ΔC_P_ΔC_R_ are based on variants of the *S. cerevisiae* Prxs Tsa2, lacking the peroxidatic (ΔC_P_) or the resolving (ΔC_R_) cysteine residues, and are sensitive to metabolic H_2_O_2_ baseline levels [93].

More roGFP-based fusion proteins have been developed for the detection of species-specific redox compounds (Figure 4b). Mrx1-roGFP2 is a fusion protein of roGFP2 and mycoredoxin-1 (Mrx-1), an oxidoreductase dependent on mycothiol, an unusual thiol compound found in the actinobacteria that functions as a redox buffer [94] (Figure 4c). Brx-roGFP2 is a fusion protein of roGFP2 and bacilliredoxin (Brx), designed for the measurement of the redox potential for bacillithiol, which works in *Staphylococcus aureus* cells as a glutathione surrogate [95,96]. Tpx-roGFP2 is a fusion protein of roGFP2 with tryparedoxin (Tpx), a Prxs, for the detection of trypanothione in *Trypanosoma* [64].

Sugiura and colleagues set up a series of sensors based on cyan FP (CFP), Sirius, mTurquois, and Venus mutants [97,98]. By inserting Cys residue couples inspired to the roGFP structure, the authors obtained two series of redox-sensitive proteins called Oba-Q (oxidation balance-sensed quenching) and Re-Q (reduction-sensed quenching), having different midpoint redox potentials applicable in different cellular environments; they generally were not pH-sensitive in physiological conditions and the different emission wavelengths consented their simultaneous use. As a drawback, however, the signal of Oba-Q and Re-Q proteins was non-ratiometric and their fluorescence intensity was proportional to their expression levels. Taking advantage of the expertise on mTurquois and Venus mutants, the same research group also conceived a FRET-based biosensor, where the two FTs were separated by the N-terminus of the Trx-targeted protein CP12 from *A. thaliana* or the cyanobacterium Anabaena sp. PCC7120 (Figure 4d) [98]. These sensors were called CROST (Change in RedOx State of Trx) 1 and 2, respectively, and could discriminate the Trx redox state directly.

Another type of sensor was then developed to monitor in living cells the glutathione redox state fluctuations through Grx activity. Indeed, Grx-FROG/B, composed of the human Grx1 domain connected by a linker to a mutant GFP, exploits a mechanism called excitation state intramolecular proton transfer (ESIPT) that gives green fluorescence in oxidizing conditions and blue emission in reducing ones, with a midpoint redox potential of −293 mV and a good response at physiological pH values (Figure 4e). The green/blue output of Grx-FROG/B can be registered at the same time by a multiplex detector [99].

### 3.3. ROS Sensors Based on Circularly Permuted FPs

Circular permutation of proteins, a naturally occurring event first described in lectins [100], consists of the modification of their primary structure by fusing the natural N- and C-termini with peptide linkers and creating new extremities, generally on exposed flexible loops (Figure 4f). Based on this approach, it is possible to insert ROS-sensitive domains inside GFP or other FP sequences, producing a chimeric structure. The native folding is easily preserved but, at the same time, it is possible to modulate FP intrinsic fluorescence through conformational rearrangements promoted by ligand binding to the receptor domain [101,102].

HyPer sensors were developed, starting in 2006, by Belousov and co-workers, by exploiting circularly permuted FPs (Figure 4g) [103,104,105,106]. A circularly permuted YFP (cpYFP) was modified to develop a chimeric construct with the regulatory domain (RD, residues 80–310) of the OxyR transcription factor from *E. coli*, specifically sensitive to H_2_O_2_ [103]. Under oxidizing conditions, OxyR switched to the DNA binding-competent form with Cys199 and Cys208 engaging in a disulfide bond. The cpYFP sequence was inserted between residues 205 and 206 of OxyR-RD by exploiting two short linkers, and the chimeric structure assembled as weak dimers [105]. This reaction required the formation of sulfenic acid derivatives between H_2_O_2_ and Cys199; Cys199 was then repelled by the hydrophobic pocket and approached Cys208 to bridge [107]. A large conformational movement, involving the flexible region between residues 205–222 on OxyR-RD, induced the cpYFP fluorescence increase [103]. Particularly, the HyPer spectrum showed two excitation peaks at 420 nm and 500 nm, and one emission peak at 516 nm. Thanks to the Y203F mutation on cpYFP, the peak at 420 nm decreased upon the reaction with H_2_O_2_, with a concomitant proportional increase in the peak at 500 nm. The ratiometric behavior of the sensor made the signal readout independent from protein concentration, and the response showed a three-fold increase in the excitation ratio of 500 nm/420 nm. HyPer was as sensitive as wild-type OxyR to H_2_O_2_ sub-micromolar concentrations, but its specificity was higher since it could not react with nitric oxide and other oxidants. In particular, in vitro tests in the presence of the purified protein reported a dynamic range between 25 nM and 250 nM, whereas the minimal H_2_O_2_ concentration revealed in vivo was 5 μM (as for wild-type OxyR), probably due to the activity of catalase and other enzymes, both in bacterial and eukaryotic cells. Very importantly, the biosensor response was reversible and, after restoring the initial fluorescence levels, several rounds of oxidation were possible. The HyPer signal, however, was pH-dependent and its utilization required the monitoring of the environment during the measurements. Also, HyPer expression could be targeted to different cellular compartments by inserting the specific localization signals, and consenting ROS measurements in selected organelles where the redox potential was compatible with that of OxyR reactive cysteine residues [108].

In the following years, an advanced version of HyPer was developed by the same research group to improve the biosensor dynamic range. The A406V mutation, corresponding to an amino acid substitution on the OxyR-RD moiety (A233V on the OxyR wild type sequence), was fortuitously discovered as improving two-fold the HyPer dynamic range; the new probe was called HyPer-2 [105]. This mutation was already characterized on *E. coli* OxyR, where it caused an apparent decreased affinity for DNA [109], while in HyPer-2 it increased the hydrophobic character of the dimerization interface, stabilizing the dimeric assembly even at low protein concentrations. The modified sensor was more sensitive to low concentrations of H_2_O_2_ in vivo than HyPer, but the half-oxidation and half-reduction times were doubled with respect to the parent probe, probably due to an altered accession to the disulfide bridge or a slow signal response related to the dimerization kinetics after oxidation [105].

To overcome the kinetic limits shown by HyPer-2, the mutation H34Y was introduced on the HyPer construct (H114Y based on wtOxyR numeration, known to give a constitutively active phenotype), originating the HyPer-3 sensor. This modification maintained the ratiometric behavior (500 nm/420 nm) of HyPer, while giving a dynamic range comparable to HyPer-2, improved the signal-to-noise ratio and the oxidation and reduction half-times of the sensor, as well as the pseudo-first-order reaction rate for H_2_O_2_, which was more than doubled with respect to the HyPer-2 probe (from 1.2 × 10^5^ M^−1^ s^−1^ for HyPer-2 to 2.5 × 10^5^ M^−1^ s^−1^ for HyPer-3). H34Y preserved the oligomeric state of HyPer-3 of a weak dimer, as already observed for the parental chimera [104].

All three HyPer sensors described here had the same quantum yield of 0.1. Despite its limitations, however, HyPer-3 had the highest extinction coefficient and was brighter than HyPer and HyPer-2, so its use was favored in the case of low expression levels [104].

In the last years, an ultrasensitive, ultrafast, and pH-stable version of HyPer, named HyPer-7, has been further developed by Belousov’s research group, greatly improving the performance and the application possibilities of the indicator [106]. The advanced probe integrated a mutated cpGFP into OxyR-RD from *Neisseria meningitidis* between residues 126 and 127. More specifically, several mutations were introduced on the YFP sequence to improve the properties of the fluorescent moiety; among them, the G298S mutation restored the chromophore triad of GFP. The use of OxyR-RD from *N. meningitidis* allowed the detection of H_2_O_2_ concentrations as low as 2 μM in vivo that could not be revealed by the HyPer sensors based on *E. coli* OxyR [106]. HyPer-7 had two excitation maxima at 400 nm and 499 nm, with a molar extinction coefficient of 74,000 M^−1^ cm^−1^, four-fold higher than HyPer-3 (17,000 M^−1^ cm^−1^), and a fluorescence quantum yield of 0.3–0.4; the sensor was therefore 15–17-fold brighter than HyPer-3. The enhanced stability of the ratiometric readout at neutral pH allowed the application of HyPer-7 in experiments involving rather large pH shifts, while the improved catalytic rate (60–86 times faster than previous sensors) granted the responsiveness necessary in real-time imaging analyses. Several derivatives of the original construct could be produced to target different cellular compartments than cytosol, such as the nucleus, mitochondria, and the mitochondrial intramembrane space; the introduction of protein binding sequences to monitor specific cellular events was also possible [106].

A modified version of HyPer, called TriPer, was conceived to measure the H_2_O_2_ fluctuations in the endoplasmic reticulum, where the oxidized protein pool of the disulfide isomerase family competed with H_2_O_2_ in reacting with HyPer, impeding its use in this compartment [110]. TriPer was obtained by mutating Ala187 (OxyR numeration) into cysteine on a HyPer sequence, a residue that was structurally proximal to Cys208; the presence of a third reactive cysteine allowed the formation of an intramolecular Cys187-Cys208 disulfide bridge that preserved a functional Cys199 fraction able to respond to H_2_O_2_, with the concomitant resolution in trans of sulfenic intermediates.

A further evolution of the HyPer sensors was represented by NeonOxIrr, a genetically-encoded sensor composed of a circularly permuted mNeonGreen FP fused to a truncated version of *E. coli* OxyR [111] via two random linkers. NeonOxIrr overcame the HyPer-3 pH sensitivity, showing a constant dynamic range between 5.5 and 7.5 pH values and an unaltered pK_a_ in the reduced and oxidized forms, as well as having a higher extinction coefficient in the oxidized state. A ratiometric version of this sensor for quantitative measurements in bacteria has been obtained by fusion with the red FP mCherry; additional improvements were later obtained for H_2_O_2_ visualization in mammalian cells and ex vivo [111].

HyPerRed, the first red fluorescent biosensor for in vivo imaging, was developed, starting from a circularly permuted mApple called cpRED obtained from the calcium sensor R-GECO1 [112]. The FP of HyPer was substituted with cpRED and optimized linkers were selected to obtain clones with efficient maturation at 37 °C. HyPerRed had an excitation peak at 575 nm, with an extinction coefficient of 39,000 M^−1^ cm^−1^, and showed a maximum emission at 605 nm; the HyPer selectivity for H_2_O_2_ was retained and the sensor sensitivity ranged between 20 nM and 300 nM in vitro, while the minimal response in *E. coli* cytoplasm was detected in the presence of 10 μM substrate. HyPerRed is an intensiometric probe without ratiometric behavior and showed a 30% lower dynamic range than HyPer; it is also pH-sensitive, and the use of a pH control system during experiments is recommended.

Another example of a red FP (RFP)-coupled biosensor was represented by rxRFP, based on the circularly permuted RFP (cpRFP) from the R-GECO1 sensor [113]. The developers inserted two cysteines at the N- and C-termini of the RFP, creating a redox-sensitive disulfide bridge responsive to GSSG, superoxide ion, and peroxynitrite; the probe was instead unreactive to H_2_O_2_, hypochlorite, and hydroxyl radical. The rxRFP spectrum showed two maximum absorption peaks at 448 nm (protonated chromophore) and 576 nm (deprotonated chromophore); the latter was responsible for the emission at 600 nm. Upon oxidation, the two bands interconverted and the switch from the reduced to the oxidized state was followed by an up to four-fold fluorescence increase. rxRFP was sensitive to pH variation during cellular measurements, and it was necessary to couple its utilization with a pH sensor indicating the environmental pH fluctuations.

rxRFP features have been exploited also by Ai’s research group to develop the first genetically encoded biosensor for Trx redox system monitoring [114]. Specifically, the optimal coupling of the redox cysteine pairs of human Trx1 and rxRFP was obtained by inserting a Gly-Ser-rich linker between the two proteins; several mutation rounds led to the set-up of TrxRFP1 (Figure 4h), which showed a fluorescence fingerprint identical to rxRFP1 alone, presenting excitation and emission maxima at 576 nm and 600 nm, respectively, with a 5.7-fold fluorescence increase in dynamic range. The result was the development of a fluorescent probe highly responsive to Trx peroxidase-mediated H_2_O_2_ oxidation, even at a nanomolar concentration; its midpoint redox potential of −281 mV makes this sensor suitable for cytoplasmic measurements in live cells where, also, TrxRFP1 is not directly oxidized by H_2_O_2_ and is not influenced by the presence of glutathione, a characteristic that consents the specific monitoring of the cellular Trx redox system [114,115].

Recently, new versions of the sensor—called TrxRFP2 and MtrxRFP2—have been developed by the same research group to improve TrxRFP1 kinetics and to create a sensor suitable for redox measurements in mitochondria [116].

The transcriptional regulator OhrR from *Xanthomonas campestris* can sense and respond to organic hydroperoxides via the oxidation of a cysteine residue, which induces a large conformational rearrangement predisposing the formation of an intermolecular disulfide bridge. Chen’s group exploited the behavior of OhrR to develop an organic hydroperoxide sensor by inserting a circularly permuted version of the fluorescent Venus protein (cpVenus) between OhrR residues 119 and 120 (Figure 4i) [117]. The probe, named OHSer (organic hydroperoxide sensor), showed a fluorescence emission at 526 nm upon excitation at 519 nm, a spectral property that adapted well to the reduction of biological damage during in vivo measurements. The authors demonstrated the high reaction selectivity of OHSer towards similar ROS in vitro, showing minimal response to H_2_O_2_ and the hydroxyl radical. Moving to in vivo measurements, the sensor expression in living cells allowed to assess in different cell lineages the absence of fluorescent background, a negligible effect of H_2_O_2_, and the detection of specific responses to cumene hydroperoxide and tert-butyl hydroperoxide.

### 3.4. ROS Sensors Based on LOV Domains

The light–oxygen–voltage-sensing domains (LOV domains) [118] are part of the PAS domain superfamily [119] (named after three proteins in which it occurs, i.e., Per, Arnt, and Sim) and are present in proteins from higher plants, microalgae, fungi, and bacteria. LOV-containing proteins are involved in the detection and adaptation to environmental changes. Particularly, LOV domains are involved in controlling phototropism, chloroplast movements, and stomatal opening in higher plants [120]. In fungi, they are involved in the circadian temporal organization of the cells. LOV domains consist of 110 to 140 amino acids organized in 5 antiparallel β-sheets and several α-helices and contain a blue-light sensitive flavin chromophore, usually flavin mononucleotide (FMN) (Figure 3c) [121]. The LOV domain of LOV-containing proteins serves as a photoswitch, activating kinase, phosphodiesterase, and DNA-binding domains upon light absorption [122]. Particularly, phototropins—blue light-activated serine/threonine protein kinases found in plants—are composed of two LOV domains (LOV1 and LOV2) and a C-terminal Ser-Thr kinase. Upon blue-light absorption, the FMN chromophore covalently binds a conserved cysteine residue. This photoreactivity mediates the activation of the kinase through autophosphorylation. Because of its small size, O_2_-independent and fast-folding LOV-based optogenetic tools have been developed [123].

The FMN chromophore of LOV domains exhibits green fluorescence when excited with UV/blue light. Upon the formation of the FMN-cysteinyl adduct in the photocycle, fluorescence is temporarily lost. Therefore, LOV-based FPs to be used as probes are based on variants lacking the reactive cysteine. The C426A LOV2 variant and its shorter and more fluorescent iLOV derivative have been employed as fluorescent reporters [25]. iLOV exhibits an excitation maximum at 477 nm and an emission centered at 497 nm, with a quantum yield of 0.44 [25].

Other than being used as fluorescent reporters, engineered LOV domains have been used to devise sensors, ranging from metals [124] to O_2_ [30]. LOV-based chimeras have also been used to develop sensors for the intracellular redox state. Among LOV-based sensors, a dual-function pH and redox-sensitive FP named pHaROS has been devised as a sensor for both H_2_O_2_ and pH in living cells. It consists of the iLOV domain fused with mBeRFP, a variant of the monomeric far-red FP mKATE (Figure 4l) [125,126]. The iLOV portion of pHaROS can reversibly gain an electron and displays fluorescence intensity changes depending on the redox state. GRX1-pHaROS is a fusion protein of pHaROS and Grx1, which confers higher redox specificity [125], in a manner similar to Grx1-roGFP2 (Figure 4m) [84,85].

### 3.5. ROS Sensors Based on YAP1

YAP1 is a transcriptional regulator that activates the transcription of genes involved in oxidative stress response and redox homeostasis in *S. cerevisiae*. YAP1, which is partially disordered, can undergo conformational changes affecting disulfide bond formation at two cysteine-rich domains (CRDs) that mask the nuclear export signal, thus preventing its nuclear export. In *S. cerevisiae* Yap1 and Orp1 constitute a redox relay system, where Yap1 is oxidized by the peroxidase Orp1 in the presence of H_2_O_2_. Two genetically encoded sensors for H_2_O_2_ based on Yap1 were reported, based on the FRET couple Cerulean Δ11 and Cp173 Venus separated by the two CRDs of Yap1 (OxyFRET, Figure 4n) [127] or by Orp1 linked with the C-terminal CRD of Yap1 (PerFRET, Figure 4o) [127]. The distance of the two FPs depends on the oxidation status of the CRD, which in turn depends on H_2_O_2_ concentration. These tools were used to measure H_2_O_2_ production by NADPH oxidases (Nox) [127].

In 2010, Yano and colleagues proposed a construct composed of the FPs cerulean and citrin linked by the cysteine-rich domain from Yap1 [128]. The biosensor, called Redoxfluor, exploited the FRET between the two FPs that allowed to calculate a fluorescence emission ratio upon excitation at 405 nm. Redoxfluor was used to explore the peroxisomal redox potential in Chinese hamster ovary (CHO) cells and to screen the effect of redox modulator agents on mutant lines.

The Yap1 binding sequence has been exploited to build a transcription factor-based biosensor in yeast that responds to H_2_O_2_, diethyl maleate, tert-butyl hydroperoxide (TBHP), and diamide [129]. This biosensor was composed of four Yap1 binding element tandem repeats, an optimized 5′-UTR, a promoter, and the mCherry reporter gene; additional controls were inserted into the yeast genome to maximize the biosensor activation [129].

### 3.6. ROS Sensors Based on Peroxiredoxin

Peroxiredoxins (Prxs) (Figure 3d) belonging to the “AhpC-Prx1” subfamily, such as mammalian Prx1 and Prx2, carry the peroxidatic and resolving cysteines on two different subunits. Upon oxidation in response to H_2_O_2_ increases, these Prx undergo a conformational rearrangement followed by dimerization. This mechanism has been used to devise a fusion-protein FRET couple with FPs perClover and mRuby2 separated by human Prx2 (Figure 4p) [130]. Upon excitation at 488 nm, the 625/525 nm emission ratio increased in function of H_2_O_2_ sensing, without the interference of pH fluctuations. The probe was highly selective toward H_2_O_2_, with the only exception of TBHP, which naturally reacted with Prxs. To characterize the response of the cytosolic probe to H_2_O_2_, HeLa cells were transfected with the probe-encoding gene, put in contact with an external bolus addition of H_2_O_2_, and imaged with a widefield microscope [130].

To obtain a Prx-based H_2_O_2_ sensor specifically devised for bacterial cells, a probe based on fungal Prxs was also produced. In this construct, a circularly permutated YPF was sandwiched between *Aspergillus nidulans* peroxiredoxin A (PrxA) and a Trx variant that can form a stable disulfide bond with Prx upon its oxidation by H_2_O_2_ [131]. The conformational change that accompanied the disulfide bond formation altered the fluorescent properties of YFP, with an increase in the excitation peak at 500 nm and a decrease in that at 415 nm [131].

## 4. Conclusions and Future Perspectives

The introduction of genetically encoded fluorescent biosensors for the monitoring of physiological processes has ushered in new fields of investigation, exemplified by the large number of published research studies. Since their first applications at the end of the 1990s, novel fluorescent biosensors have seen continuous development and improvement. The development of FPs excitable at different wavelengths and the insertion of signal peptides has allowed for multiplex imaging and detection in different cellular compartments. 

In recent years, advancements and cost reductions in the genetic engineering field have made it possible to establish and study complex systems that offer greater insights into physio-pathological phenomena. Great support has also derived from the important progress made at the detection level, with the development of optical instruments with improved signal registration, reduced scattering and photobleaching effects, and quantitative readouts. In this context, fluorescent biosensors that yield ratiometric signals and are not pH-dependent are the base for robust and reproducible measurements. Moreover, broadening the biosensor panel to near-infrared probes would offer new excitation wavelengths and allow to reach deeper tissues in vivo. Altogether, these advancements will enable the attainment of high spatiotemporal resolution in the field of redox biology.

## Data Availability

Not applicable.
